# An observational study on lupus nephritis combined with cryoglobulinemia

**DOI:** 10.1080/0886022X.2026.2647568

**Published:** 2026-04-06

**Authors:** Yao Yang, Meng Tan, Xin Zhang, Minghui Zhao, Ying Tan

**Affiliations:** ^a^ Renal Division, Department of Medicine, Peking University First Hospital, Institute of Nephrology, Peking University, Beijing, China; ^b^ Key Laboratory of Renal Disease, Ministry of Health of China, Key Laboratory of Chronic Kidney Disease Prevention and Treatment, Ministry of Education of China, Beijing, China; ^c^ Research Units of Diagnosis and Treatment of Immune-Mediated Kidney Diseases, Chinese Academy of Medical Sciences, Beijing, China; ^d^ Department of Nephrology, NHC Key Laboratory of Pulmonary Immunological Disease, Guizhou Provincial Key Laboratory of Pathogenesis and Prevention of Common Chronic Diseases Research, Guizhou Provincial People’s Hospital, Guiyang, China

**Keywords:** Systemic lupus erythematosus, lupus nephritis, cryoglobulinemia, plasma exchange

## Abstract

Lupus nephritis (LN) is a common complication of systemic lupus erythematosus (SLE), and cryoglobulinemia is relatively common in SLE, with both conditions potentially leading to kidney injury. This study aimed to describe the clinical features of LN with cryoglobulinemia and explore whether more aggressive treatment should be considered in such cases. This retrospective study at Peking University First Hospital (2006–2024) analyzed 127 LN patients screened for cryoglobulinemia to assess its prevalence, clinical impact, and treatment implications. 63.8% of LN patients showed concurrent cryoglobulinemia. Patients with cryoglobulinemia showed more severe hypocomplementemia (*p* = 0.002) and leukocyturia (*p* = 0.018). Type II cryoglobulinemia (*n* = 9) showed higher serum creatinine (*p* = 0.025) and IgM (*p* = 0.030) versus those with type III (*n* = 70). 13 out of 81 patients with cryoglobulinemia exhibited ultrastructural changes on electron microscopy. The average follow-up was 58.0 (39.0, 87.0) months; 58.0% achieved complete renal response (CRR), 25.9% attained partial renal response (PRR), and 16.1% had no renal response. After propensity score matching (PSM), patients with cryoglobulinemia who received plasma exchange (PE) therapy showed no statistically significant difference in renal remission compared to those who did not undergo PE therapy (*p* = 1.000). Cox regression showed that cryoglobulinemia was not an independent risk factor of poor renal outcomes in LN (HR = 1.004, 95% CI [0.498–2.021], *p* = 0.992). Cryoglobulinemia is prevalent and was not an independent risk factor for renal prognosis in LN. PE may not provide additional benefits in LN patients with cryoglobulinemia.

## 1. Introduction

Systemic lupus erythematosus (SLE) is a common autoimmune disease of unknown cause that primarily affects women of childbearing age. Its clinical manifestations are heterogeneous and can lead to damage in various tissues and organs, with the kidneys being one of the most vulnerable organ tissues to SLE. Lupus nephritis (LN) is a complication of SLE that significantly affects the incidence and overall mortality rates of SLE [1]. Nearly all LN patients have antinuclear antibodies (ANA), and most have anti-double-stranded DNA (dsDNA) antibodies, along with hypocomplementemia [2]. Among them, approximately 19–83% have a positive cryoglobulin test [3–6].

Cryoglobulins are immunoglobulins that precipitate reversibly at low temperatures. In cold temperatures, these proteins clump together and block blood vessels, potentially causing a wide range of complications and affecting multiple systems including the kidneys [7,8]. In 1974, Brouet and colleagues established a classification of cryoglobulinemia based on the immunoglobulin type, which is still used today: type I consists of a monoclonal immunoglobulin (IgM, IgG, or IgA), type II includes polyclonal IgG plus monoclonal IgM with rheumatoid-factor activity, and type III comprises polyclonal IgG, polyclonal IgM, or both. Both type II and type III are known as mixed cryoglobulinemia [9]. Mixed cryoglobulinemia (including types II and III) is associated with B-cell lymphoproliferative diseases, chronic infections (mainly hepatitis C virus, HCV), and autoimmune diseases. SLE is one of the most common autoimmune diseases associated with cryoglobulinemia, though many patients with cryoglobulinemia may remain asymptomatic [5,6,10,11].

Currently, there is limited data on patients with SLE and concurrent cryoglobulinemia, especially the influence on kidneys. The clinical impact of cryoglobulinemia on LN remains poorly defined. Therefore, our study aims to describe the clinical and biological characteristics of LN patients with cryoglobulinemia and to explore whether more intensive therapy should be considered in the presence of cryoglobulinemia in LN patients.

## 2. Materials and methods

### 2.1. Patients

The clinical and renal histopathological data of patients diagnosed with lupus nephritis (LN) at Peking University First Hospital from February 2006 to January 2024 were reviewed. Patients were included for the study based on the following criteria: (1) met the 1997 American College of Rheumatology revised criteria or the 2019 ACR/European League Against Rheumatism (EULAR) classification criteria [12,13], (2) diagnosis of LN by kidney biopsy; (3) qualitative and classification tests for cryoglobulins were performed. Exclusion criteria include patients who did not undergo cryoglobulin testing, hepatitis C virus (HCV)-infected or hepatitis B virus (HBV), and patients without follow-up records. The patients were classified according to their cryoglobulin status. The pathological sections were reviewed by two pathologists, excluding other types of glomerulonephritis.

Each patient signed the informed consent form regarding clinical data collection, blood sample collection, renal biopsy, and follow-up examinations, thereby obtaining written consent from each patient. The research was in compliance with the Declaration of Helsinki and was approved by the ethics committee of Peking University First Hospital (approval number: 2025-yan187).

### 2.2. Clinical data collection

Clinical data were extracted from the electronic medical records of Peking University First Hospital, including clinical manifestations, levels of serum creatinine, complete blood cell count, 24-h proteinuria, hematuria, leukocyturia and cylindruria, antinuclear antibodies, anti-dsDNA antibodies, serum C3 and C4. Disease activity was assessed using the Systemic Lupus Erythematosus Disease Activity Index 2000 score (SLEDAI-2K). The renal biopsy specimens were examined by light microscopy, direct immunofluorescence, and electron microscopy techniques. Patients or the public were not involved in the design, conduct, or reporting of our study.

The cryoglobulin tests were determined using the routine procedures of the Peking University First Hospital Laboratory. Blood samples were obtained and kept at 37 °C for 30 min before separation. Serum was prepared by centrifuging for 15 min at 2000 rpm. Fresh centrifuged serum was incubated at 4 °C for 72 h to seven days after collection and examined for cryoprecipitate. The cryocrit was obtained by centrifuging at 2,000 rpm (750 g) for 30 min at 4 °C. The cryoprecipitate was diluted in warm saline for one hour. Finally, dissolved cryoprecipitate was identified by agarose gel electrophoresis and immunofixation.

The viral status against HBV, HCV, and human immunodeficiency virus (HIV) was also registered. HBV status was considered positive if HBsAg antigen was present, and HCV status was considered positive if anti-HCV antibodies were present, regardless of the viral load status [14,15].

The complete renal response (CRR) was defined as 24h proteinuria <0.5 g/d, stabilization or improvement in renal function (0 ± 15% of baseline). The partial renal response (PRR) was defined as a reduction in proteinuria at least 50% and to <3 g/d, stabilization or improvement in renal function (0 ± 15% of baseline). No renal response was defined as failure to achieve PRR or CRR [16]. The primary endpoint is defined as end-stage renal disease (eGFR < 15 mL/min·1.73m^2^) or death, and the secondary endpoint is defined as a serum creatinine increase of 20% or more from baseline.

### 2.3. Statistical analysis

Statistical analyses were performed using SPSS 27 (SPSS Inc., Chicago, IL, USA) and R, version 4.4.3 (R Foundation for Statistical Computing, Vienna, Austria). Continuous data are presented as mean ± standard deviation (SD) and compared using Student’ s t-test for normally distributed variables. Non-normally distributed variables are presented as interquartile ranges (IQR) and compared using the Mann-Whitney U test. Categorical variables are expressed as proportions and compared using the chi-square test. Kaplan-Meier curves were used to analyze patient prognosis. Univariate and multivariate COX regression analysis was used to evaluate renal prognostic factors. Results were expressed as HR with 95% CIs. Using 1:1 nearest-neighbor matching with a caliper restriction set at 0.2 log units of the standard difference, a propensity score-matched (PSM) cohort was established incorporating age, sex, and serum creatinine. All *P* values are two-sided, and statistical significance was defined as *p* < 0.05.

## 3. Results

### 3.1. Clinical and laboratory features in the whole cohort

Between 2006 and 2024, a total of 526 biopsy-proven LN patients were screened, and 127 LN patients were included in the study (flowchart in Supplementary Figure S1). Based on cryoglobulin test results at baseline, 81 patients were enrolled as group of LN patients with cryoglobulinemia, while the remaining 46 patients comprised control group of ones without cryoglobulinemia.

In the cohort, 91 (71.7%) patients were female, median age was 30.0 (25.0, 46.0) years, and the median follow-up duration was 58.0 (39.0, 87.0) months. By the time of enrollment, the median range of SLEDAI-2K was 16.0 (12.0, 20.0). Over 90% of patients (119/127) presented with proteinuria (UTP ≥ 0.5 g/d), median levels of serum creatinine at baseline were 84.0 (63.1, 174.7) μmol/L, and 40 patients (31.5%) experienced acute kidney disease (AKD) at the onset of lupus when they sought medical care at our hospital. During the follow-up period, five patients died, and twelve patients progressed to end-stage kidney disease (ESKD).

Compared to the control group, LN patients with cryoglobulinemia exhibited higher proportions of leukocyturia (63.0% vs 41.3%, *p =* 0.018), low complement levels (C3 0.389 (0.303, 0.498) vs 0.481 (0.388, 0.658) g/L, *p* = 0.002; C4 0.064 (0.038, 0.116) vs 0.124 (0.078, 0.178) g/L, *p <* 0.001) and a lower incidence of thrombocytopenia (3.7% vs 21.7%, *p* = 0.002) (detailed in Table 1).

**Table 1.** Comparisons of clinicopathological data of LN patients with and without cryoglobulinemia.

	LN with cryoglobulinemia (*n* = 81)	LN without cryoglobulinemia (*n* = 46)	P value
Sex/female, n (%)	60 (74.1%)	31 (67.4%)	0.422
Age at diagnosis of SLE, years	28.0 (23.0, 40.5)	32.0 (26.0, 48.5)	0.060
Clinical manifestations			
Visual disturbance, n (%)	3 (3.7%)	0	0.553
Arthritis, n (%)	8 (9.9%)	6 (13.0%)	0.584
Rush, n (%)	23 (28.4%)	11 (23.9%)	0.583
Alopecia, n (%)	13 (16.0%)	5 (10.9%)	0.421
Mucosal ulcers, n (%)	5 (6.2%)	3 (6.5%)	1.000
Fever, n (%)	5 (6.2%)	2 (4.3%)	1.000
Raynaud’s phenomenon, n (%)	3 (3.7%)	1 (2.2%)	1.000
NPSLE, n (%)	0	0	
Renal features			
Urinary casts, n (%)	33 (40.7%)	12 (26.1%)	0.097
Hematuria, n (%)	63 (77.8%)	37 (80.4%)	0.725
Proteinuria, n (%)	76 (93.8%)	43 (93.5%)	1.000
Leukocyturia, n (%)	51 (63.0%)	19 (41.3%)	**0.018**
AKD, n (%)	25 (30.9%)	15 (32.6%)	0.839
NS, n (%)	46 (56.8%)	23 (50%)	0.460
Laboratory tests			
WBC,10^9^/L (3.5–9.5 109/L)	5.76 (3.85, 8.05)	5.60 (3.68, 9.40)	0.910
Hb, g/L (115–150 g/L)	105.0 (90.0, 121.0)	103.5 (81.0, 120.3)	0.748
PLT,10^9^/L (125–350 109/L)	189.0 (132.0, 262.0)	204.5 (123.0, 250.8.0)	0.902
Alb, g/L (40–55 g/L)	27.17 ± 6.55	27.22 ± 6.83	0.965
Scr, μmol/L (44–133 μmol/L)	84.7 (60.8, 166.6)	82.0 (64.7, 209.8)	0.416
24-hour urinary protein, g/day (0–0.15 g/24h)	3.88 (1.88, 6.25)	3.96 (1.65, 9.07)	0.319
C3, g/L (0.6–1.5 g/L)	0.389 (0.303, 0.498)	0.481 (0.388, 0.658)	**0.002**
C4, g/L (0.12–0.36 g/L)	0.064 (0.038, 0.116)	0.124 (0.078, 0.178)	<0.001
IgG, g/L (7.23–16.85 g/L)	12.00 (7.06, 16.40)	11.30 (5.69, 14.40)	0.150
IgA, g/L (0.69–3.82 g/L)	2.20 (1.76, 3.29)	2.32 (1.73, 3.37)	0.904
IgM, g/L (0.63–2.77 g/L)	0.95 (0.66, 1.36)	0.73 (0.47, 1.30)	0.056
ANA, n (%)	81 (100%)	43 (93.5%)	0.113
Anti-ds-DNA antibody, n (%)	58 (71.6%)	27 (58.7%)	0.137
Anti-Sm antibody (+), n (%)	18 (22.2%)	6 (13.0%)	0.204
Anti-rRNP antibody(+), n (%)	20 (24.7%)	5 (10.9%)	0.060
SLEDAI-2K	16.5 ± 5.8	15.4 ± 6.9	0.320
Renal pathology			
Class I, n (%)	1 (1.2%)	0	0.531
Class II, n (%)	3 (3.7%)	0	
Class III (include III+V), n (%)	13 (16.0%)	10 (21.7%)	
Class IV (include IV+V), n (%)	53 (65.4%)	27 (58.7%)	
Pure Class V, n (%)	11 (13.6%)	9 (19.6%)	
Activity indices score	8.0 (5.0, 11.0)	7.0 (3.8, 10.0)	0.398
Endocapillary hypercellularity	3.0 (1.0, 3.0)	2.5 (1.0, 3.0)	0.397
Glomerular leukocyte infiltration/Karyorrhexis	1.0 (1.0, 3.0)	1.0 (0, 2.0)	0.181
Fibrinoid necrosis	0 (0, 0)	0 (0, 0)	0.370
Cellular crescents	2.0 (0, 2.0)	2.0 (0, 2.0)	0.609
Platinum ear/microthrombosis	1.0 (0, 2.0)	0 (0, 1.0)	0.252
Interstitial inflammatory cell infiltration	1.0 (0, 1.5)	1.0 (1.0, 1.3)	0.337
Chronicity indices score	1.0 (0, 2.0)	1.5 (0, 3.0)	0.484
Glomerular sclerosis	0 (0, 1.0)	0 (0, 1.0)	0.207
Fibrous crescents	0 (0, 0)	0 (0, 0)	0.414
Tubular atrophy	1.0 (0, 1.0)	1.0 (0, 1.0)	0.571
Interstitial fibrosis	0 (0, 1.0)	1.0 (0, 1.0)	0.563
Associated autoimmune disease			
APS, n (%)	4 (4.9%)	3 (6.5%)	0.703
Sjögren’s syndrome, n (%)	3 (3.7%)	2 (4.3%)	1.000
Therapeutic options			
High-dose GCs, n (%)	35 (43.2%)	19 (41.3%)	0.835
CTX, n (%)	46 (56.8%)	30 (65.2%)	0.309
MMF, n (%)	20 (24.7%)	9 (19.6%)	
CNIs, n (%)	12 (14.8%)	3 (6.5%)	
Others (include LEF), n (%)	3 (3.7%)	4 (8.7%)	
PE, n (%)	7 (8.6%)	4 (4.9%)	1.000
Rituximab, n (%)	2 (2.5%)	2 (4.3%)	0.620
Belimumab, n (%)	6 (7.4%)	1 (2.2%)	0.421
Follow-up time, months	61.0 (42.5, 88.0)	47.0 (36.8, 82.3)	0.125
Renal remission status			
CRR, n (%)	47 (58.0%)	22 (47.8%)	0.501
PRR, n (%)	21 (25.9%)	16 (34.8%)	
NR, n (%)	13 (16.1%)	8 (17.4%)	
Long-term survival			
Died, n (%)	4 (4.9%)	1 (2.2%)	0.653
ESKD, n(%)	7 (8.6%)	5 (10.9%)	0.756

Abbreviations: LN, lupus nephritis; SLE, systemic lupus erythematosus; AKD, acute kidney disease; NS, nephrotic syndrome; WBC, white blood cell; Hb, Hemoglobin; PLT, Platelet; Alb, albumin; Scr, serum creatinine; C3, Complement 3; C4, Complement 4; IgG, Immunoglobulin G; IgA, Immunoglobulin A; IgM, Immunoglobulin M; ANA, antinuclear antibody; Anti ds-DNA, anti-double-stranded DNA antibody; Anti Sm, anti-smith antibody; anti rRNP, anti-ribonucleoprotein antibody; APS, antiphospholipid syndrome; SLEDAI-2K, systemic lupus erythematosus disease activity index 2000; GCs, glucocorticoids; CTX, cyclophosphamide; MMF, mycophenolate mofetil; CNIs, calcineurin inhibitors; LEF, leflunomide; PE, plasma exchange; CRR, complete renal response; PRR, partial renal response; NR, no response; ESKD, end stage kidney disease.

### 3.2. Characteristics of cryoglobulin in subgroup of LN patients with cryoglobulinemia

In LN patients with cryoglobulinemia, the majority of cryoglobulin was type III (86.4%, 70/81). Of these, 39.5% (32/81) had polyclonal IgG, IgM, and IgA, 21.0% (17/81) had polyclonal IgG and IgM, and 21.0% (17/81) had only polyclonal IgG. A smaller proportion of patients had type II cryoglobulin (11.1%, 9/81), and 2.5% (2/81) had insufficient protein content for typing. Notably, no type I cryoglobulins were detected in our cohort.

Compared to the type III cryoglobulinemia group, type II cryoglobulinemia group exhibited significantly higher serum creatinine levels 141.0 (95.3, 273.3) vs 80.1 (59.4.0, 143.2) μmol/L, *p* = 0.025) and IgM concentrations (1.40 (0.82, 2.24) vs 0.92 (0.64, 1.24) mg/L, *p* = 0.030) than the type III cryoglobulinemia group. No significant differences in other clinical features between LN patients with different types of cryoglobulinemia (detailed in Table 2).

**Table 2.** Comparisons of clinicopathological data of LN patients’ different types of cryoglobulinemia.

	LN with type II cryoglobulinemia (*n* = 9)	LN with type III cryoglobulinemia (*n* = 70)	P value
Sex/female, n (%)	7 (77.8%)	52 (74.3%)	1.000
Age at diagnosis of SLE, years	28.0 (22.0, 45.0)	28.5 (23.0, 41.5)	0.945
Renal features			
Urinary casts, n (%)	3 (33.3%)	30 (42.9%)	0.727
Hematuria, n (%)	4 (44.4%)	29 (41.4%)	1.000
Proteinuria, n (%)	8 (88.9%)	53 (75.7%)	0.676
Leukocyturia, n (%)	8 (88.9%)	66 (94.3%)	0.463
AKD, n (%)	5 (55.6%)	19 (27.1%)	0.121
NS, n (%)	3 (33.3%)	42 (60.0%)	0.163
Laboratory tests			
WBC, 109/L (3.5–9.5 109/L)	5.52 (3.27, 13.15)	5.53 (3.88, 7.83)	0.740
Hb, g/L (115–150 g/L)	92.6 ± 24.1	106.8 ± 21.3	0.067
PLT, 109/L (125–350 109/L)	141.0 (116.0, 198.5)	191.0 (136.3, 269.8)	0.110
Alb, g/L (40–55 g/L)	27.8 ± 3.4	26.9 ± 6.8	0.533
Scr, μmol/L (44–133 μmol/L)	141.0 (95.3, 273.3)	80.1 (59.4.0, 143.2)	**0.025**
24-hour urinary protein, g/day (0–0.15 g/24h)	3.34 (1.46, 3.93)	4.33 (1.89, 7.09)	0.107
C3, g/L (0.6–1.5 g/L)	0.387 (0.230, 0.477)	0.395 (0.311, 0.490)	0.327
C4, g/L (0.12–0.36 g/L)	0.065 (0.029, 0.117)	0.062 (0.040, 0.116)	0.654
IgG, g/L (7.23–16.85 g/L)	12.40 (8.26, 18.20)	11.95 (6.97, 16.15)	0.594
IgA, g/L (0.69–3.82 g/L)	2.13 (1.61, 3.97)	2.30 (1.77, 3.28)	0.920
IgM, g/L (0.63–2.77 g/L)	1.40 (0.82, 2.24)	0.92 (0.64, 1.24)	**0.030**
SLEDAI-2K	18.3 ± 6.2	16.4 ± 5.7	0.349
Renal pathology			
Class I, n (%)	0	1 (1.4%)	0.733
Class II, n (%)	0	3 (4.3%)	
Class III (include III+V), n (%)	2 (22.2%)	11 (15.7%)	
Class IV (include IV+V), n (%)	7 (77.8%)	44 (62.9%)	
Pure Class V, n (%)	0	11 (15.7%)	
Activity indices score	9.0 (5.5, 13.0)	8.0 (4.0, 11.0)	0.259
Endocapillary hypercellularity	3.0 (1.5, 3.0)	3.0 (1.0, 3.0)	0.485
Glomerular leukocyte infiltration/Karyorrhexis	2.0 (1.0, 3.0)	1.0 (1.0, 3.0)	0.184
Fibrinoid necrosis	0 (0, 0)	0 (0, 0)	0.592
Cellular/fibrocellular crescent	2.0 (2.0, 4.0)	0.5 (0, 2.0)	0.076
Platinum ear/microthrombosis	1.0 (0, 2.0)	1.0 (0, 2.0)	0.799
Interstitial inflammatory cell infiltration	1.0 (1.0, 2.0)	1.0 (0, 1.0)	0.292
Chronicity indices score	2.0 (0.5, 3.0)	1.0 (0, 2.0)	0.371
Glomerular sclerosis	0 (0, 0.5)	0 (0, 1.0)	0.791
Fibrous crescents	0 (0, 0.5)	0 (0, 0)	0.215
Tubular atrophy	1.0 (0, 1.0)	0 (0, 1.0)	0.686
Interstitial fibrosis	1.0 (0, 1.0)	0 (0, 1.0)	0.202
Associated autoimmune disease			
APS, n (%)	0	4 (5.7%)	1.000
Sjögren’s syndrome, n (%)	2 (22.2%)	1 (1.4%)	**0.033**
Therapeutic options			
High-dose GCs, n (%)	6 (66.7%)	29 (41.4%)	0.440
CTX, n (%)	7 (77.8%)	38 (54.3%)	0.507
MMF, n (%)	2 (22.2%)	18(25.7%)	
CNIs, n (%)	0	12 (17.1%)	
Others (included LEF), n(%)	0	2 (2.9%)	
PE, n (%)	3(33.3%)	4 (5.7%)	**0.029**
Rituximab, n (%)	0	2 (2.9%)	1.000
Belimumab, n (%)	0	6 (8.6%)	1.000
Follow-up time, months	68.0 (21.5, 90.0)	61.0 (44.8, 88.0)	0.422
Renal remission status			
CRR, n (%)	6 (66.7%)	40 (57.1%)	0.552
PRR, n (%)	1 (11.1%)	20 (28.6%)	
NR, n (%)	2 (22.2%)	10 (14.3%)	
Long-term survival			
Died, n (%)	2 (22.2%)	1 (1.4%)	**0.033**
ESKD, n(%)	1 (11.1%)	6 (8.6%)	0.586

Abbreviations: LN, lupus nephritis; SLE, systemic lupus erythematosus; AKD, acute kidney disease; NS, nephrotic syndrome; WBC, white blood cell; Hb, Hemoglobin; PLT, Platelet; Alb, albumin; Scr, serum creatinine; C3, Complement 3; C4, Complement 4; IgG, Immunoglobulin G; IgA, Immunoglobulin A; IgM, Immunoglobulin M; SLEDAI-2K, systemic lupus erythematosus disease activity index 2000; APS, antiphospholipid syndrome; GCs, glucocorticoids; CTX, cyclophosphamide; MMF, mycophenolate mofetil; CNIs, calcineurin inhibitors; LEF, leflunomide; PE, plasma exchange; CRR, complete renal response; PRR, partial renal response; NR, no response; ESKD, end stage kidney disease. Bold values indicate statistical significance, meaning the corresponding *P*-value is less than 0.05.

### 3.3. Renal pathological data

In the whole cohort, renal histopathology showed that 23 patients (18.1%) had Class III LN (including Class III+V), 80 patients (63.0%) had Class IV LN (including Class IV+V), and 20 patients (15.7%) had pure Class V LN. More than 80% of the patients (103 patients) had proliferative lesions on renal biopsy. The median activity index score was 8.0 (4.0, 10.0), and the median chronicity index score was 1.0 (0, 3.0).

In LN patients with cryoglobulinemia, renal histopathology revealed that 16% (13 patients) had Class III LN (including Class III+V), 65.4% (53 patients) had Class IV LN (including Class IV+V), and 13.6% (11 patients) had pure Class V LN. Immunofluorescence studies demonstrated that none of the patients with type II cryoglobulinemia exhibited monoclonal immunoglobulin deposition. No significant pathological abnormalities were observed between the group with cryoglobulinemia and the control group (Table 1).

In patients with cryoglobulinemia, 16.0% (13/81) exhibited ultrastructural changes in the glomerular epithelium and subendothelium under electron microscopy. These alterations were characterized by microtubular structures in two patients, fibrillar structures in one patient, fingerprint-like structures in nine patients, and tubular diffuse structures in one patient. LN patients with ultrastructural changes were diagnosed with SLE at an earlier age compared to those without changes (Age at diagnosis of SLE, 26.0 (16.5, 29.5) vs 30.0 (24.3, 45.5) years, *p* = 0.024). However, there were no significant differences between the two groups regarding other clinical symptoms, immunological markers, or pathological features (detailed in Supplementary Table S1).

### 3.4. Treatment and prognosis

Among all 81 the LN patients with cryoglobulinemia, the 80 patients received timely induction therapies with glucocorticoids and immunosuppressants upon diagnosis of LN (with SLEDAI-2K scores of ≥ 6), including 35 patients received pulse steroid therapy and 7 patients received plasma exchanges (PE) at acute phase of lupus nephritis. The immunosuppressants were cyclophosphamide (*n* = 46), mycophenolate mofetil (*n* = 20), calcineurin inhibitors (*n* = 12), or leflunomide (*n* = 1). Compared with LN patients negative for cryoglobulin, although those patients with cryoglobulinemia received more PE (*n* = 7) and B-cell-targeted therapies (2 rituximab and 6 belimumab), there were no significant differences in induction therapies between the two groups.

The median follow-up duration was 58.0 (39.0, 87.0) months, among the patients with cryoglobulinemia, 58.0% of patients achieved complete renal response (CRR), 25.9% of patients achieved partial renal response (PRR), 16.1% of patients had no renal response. Among these, 7 patients progressed to end-stage kidney disease (ESKD), and an additional 4 patients died. There was no significant difference in survival prognosis between the LN group with cryoglobulinemia. Factors with *p* < 0.05 in univariate analysis (including proteinuria, leukocyturia, anti-dsDNA positivity, undergoing PE therapy, IgA, chronicity index score, glomerular sclerosis, tubular atrophy, interstitial fibrosis, and SLEDAI-2K score), along with sex, age, and cryoglobulin positivity, were included in the COX regression analysis. The results demonstrated that the presence of cryoglobulinemia was not a significant risk factor for renal prognosis in patients with LN (HR = 0.510, 95% CI [0.23–1.13], *p* = 0.097) (Supplementary Table S2).

**Figure 1.**
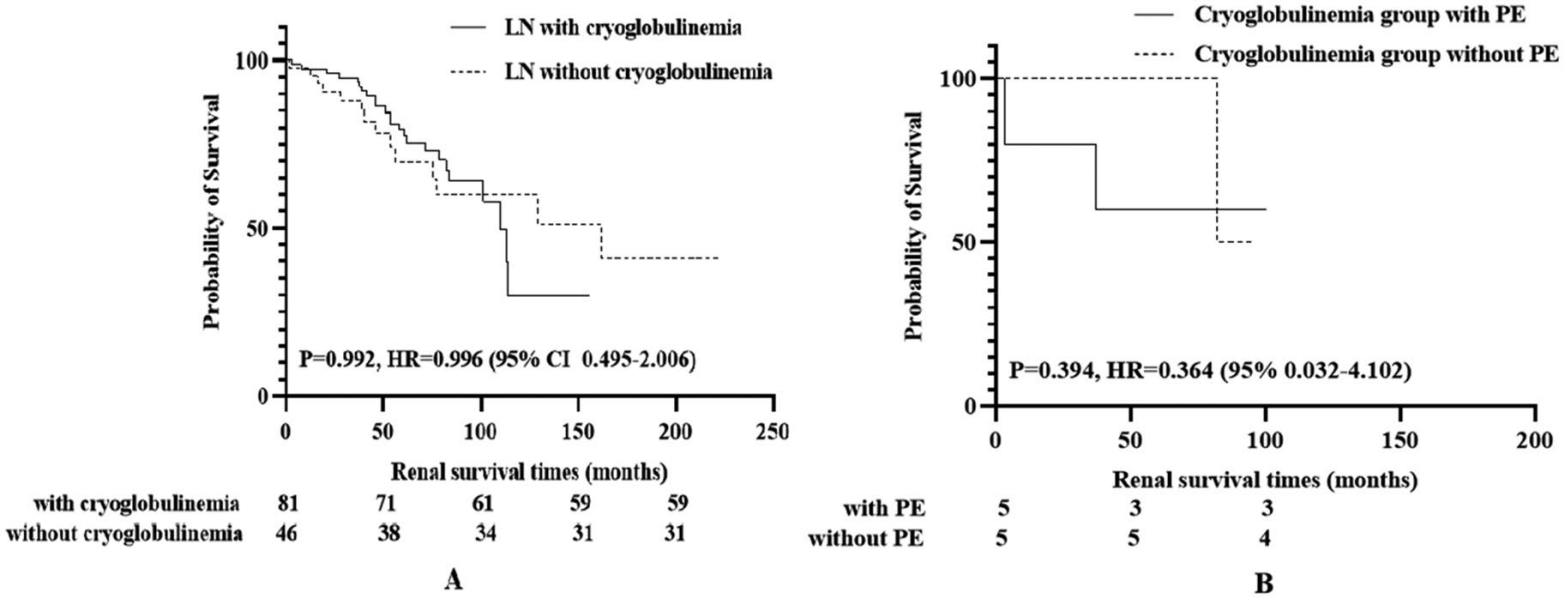
Kaplan-Meier survival analysis of composite endpoint events. *Notes*: A. Kaplan-Meier analysis of composite endpoints in LN patients with and without cryoglobulinemia. B. Kaplan-Meier analysis of the composite endpoint in matched cohorts of cryoglobulinemia-positive LN patients with versus without plasma exchange therapy.

In our cohort of cryoglobulinemia-positive LN patients, 7 patients received PE therapy. Among them, 4 patients (57.1%) achieved complete/partial renal remission, while 3 patients (42.9%) progressed to ESKD. A 1:1 propensity score matching (PSM) cohort was established by incorporating age, sex, and serum creatinine levels to mitigate selection bias. The study population included 10 LN patients with cryoglobulinemia, who were further divided into subgroups receiving PE therapy (*n* = 5) and non-PE therapy (*n* = 5). The two groups showed no significant differences in renal clinical symptoms, laboratory tests, renal pathology, or induction therapy regimens, demonstrating comparability. Ultimately, there were no statistically significant differences between the two cohorts in terms of renal remission (CR 60.0% vs 80.0%, *p* = 1.000) or the incidence of endpoint events (EKRD 40.0% vs 20.0%, *p* = 1.000). The detailed results are presented in Table 3 and Figure 1.

**Table 3.** Comparisons of renal outcomes in the group with cryoglobulinemia underwent PE therapy and those without PE.

	Cryoglobulinemia positive group with PE	Cryoglobulinemia positive group without PE	P value
Number of patients	5	5	
Sex/female, n (%)	4 (80.0%)	4 (80.0%)	1.000
Age at diagnosis of SLE, years	29.8 ± 13.3	24.8 ± 4.2	0.446
Renal manifestations			
Urinary casts, n (%)	3 (60.0%)	2 (40.0%)	1.000
Hematuria, n (%)	2 (40.0%)	4 (80.0%)	0.524
Proteinuria, n (%)	5 (100.0%)	5 (100.0%)	1.000
Leukocyturia, n (%)	3 (60.0%)	2 (40.0%)	1.000
AKD, n (%)	4 (80.0%)	4 (80.0%)	1.000
NS, n (%)	4 (80.0%)	5 (100.0%)	1.000
Laboratory tests			
WBC, 10^9^/L (3.5–9.5 10^9^/L)	8.44 ± 5.54	8.95 ± 2.60	0.856
Hb, g/L (115–150 g/L)	79.0 (62.5, 88.5)	94.0 (84.5, 96.5)	0.142
PLT, 10^9^/L (125–350 10^9^/L)	162.0 ± 65.6	214.0 ± 123.2	0.429
Alb, g/L (40–55 g/L)	26.28 ± 4.29	24.04 ± 3.98	0.417
Scr, μmol/L (44–133 μmol/L)	233.1 ± 55.3	204.5 ± 65.9	0.479
24-hour urinary protein, g/day (0–0.15 g/24h)	5.03 ± 2.57	8.65 ± 5.23	0.202
SLEDAI-2K	21.0 ± 2.2	19.0 ± 4.7	0.424
Renal pathology			
Class IV (include IV+V), n (%)	5 (100.0%)	5 (100.0%)	1.000
Activity indices score	14.0 (11.5, 14.0)	10.0 (9.5, 15.0)	0.654
Endocapillary hypercellularity	3.0 (3.0, 3.0)	3.0 (3.0, 3.0)	1.000
Glomerular leukocyte infiltration/Karyorrhexis	3.0 (2.0, 3.0)	2.0 (1.5, 3.0)	0.343
Fibrinoid necrosis	0 (0, 0)	0 (0, 2.0)	0.134
Cellular/fibrocellular crescent	6.0 (1.0, 6.0)	2.0 (2.0, 3.0)	0.309
Platinum ear/microthrombosis	1.4 ± 1.3	2.0 ± 0.7	0.402
Interstitial inflammatory cell infiltration	2.0 (1.5, 2.5)	1.0 (1.0, 2.0)	0.166
Chronicity indices score	3.0 ± 2.3	1.6 ± 1.1	0.277
Glomerular sclerosis	0 (0, 1.5)	0 (0, 1.0)	0.811
Fibrous crescents	0 (0, 1.0)	0 (0, 0)	0.134
Tubular atrophy	1.0 (0, 2.0)	1.0 (0, 1.0)	0.502
Interstitial fibrosis	1.0 (0.5, 1.5)	1.0 (0, 1.0)	0.339
Therapeutic options			
High-dose GCs, n (%)	5 (100.0%)	4 (80.0%)	1.000
CTX, n (%)	4 (80.0%)	4 (80.0%)	1.000
MMF, n (%)	1 (20.0%)	1 (20.0%)	
Rituximab, n (%)	0	0	1.000
Belimumab, n (%)	1 (20.0%)	0	1.000
Follow-up time, months	60.6 ± 24.8	69.0 ± 26.9	0.622
Renal remission status			
CRR, n (%)	3 (60.0%)	4 (80.0%)	1.000
PRR, n (%)	0	0	
NR, n (%)	2 (40.0%)	1 (20.0%)	
Long-term survival			
Died, n (%)	1 (20.0%)	0	1.000
ESKD, n(%)	2 (40.0%)	1 (20.0%)	1.000

Abbreviations: AKD, acute kidney disease; NS, nephrotic syndrome; WBC, white blood cell; Hb, Hemoglobin; PLT, Platelet; Alb, albumin; Scr, serum creatinine; SLEDAI-2K, systemic lupus erythematosus disease activity index 2000; CTX, cyclophosphamide; MMF, mycophenolate mofetil; CNIs, calcineurin inhibitors; LEF, leflunomide; PE, plasma exchange; CRR, complete renal response; PRR, partial renal response; NR, no response; ESKD, end stage kidney disease.

## 4. Discussion

Approximately 40% of noninfectious mixed cryoglobulinemia is associated with autoimmune diseases, especially SLE and Sjögren’s syndrome are more common [17]. There is relatively little research on the clinical significance of cryoglobulinemia in SLE and LN. In particular, the clinical significance and treatment strategies for cryoglobulinemia in the context of LN remain unclear. Therefore, this study primarily investigates the renal manifestations, and clinical outcomes of LN patients with concomitant cryoglobulinemia, and explores whether more aggressive treatment strategies should be adopted for these patients.

Our study found that 63.8% (81/127) of patients had at least one positive cryoglobulin test in their medical history, with this detection rate being higher than the data reported in some previous studies but similar to studies by Roubertou et al. [5,6,10]. Possible reasons for this phenomenon include significant improvements in laboratory techniques for cryoglobulin detection [18]. Simultaneously, we observed that leukocyturia was more prevalent in LN patients with cryoglobulinemia, although the underlying mechanisms remain unclear. This may be the result of dual autoimmune injury and synergistic amplification of inflammation. The monoclonal or polyclonal immunoglobulins of cryoglobulins and autoimmune complexes of SLE (such as anti-dsDNA antibodies) deposit in renal tissues, leading to the infiltration of inflammatory cells such as neutrophils and monocytes into the kidneys. At the same time, the deposition of cryoglobulin immunoglobulins and lupus nephritis-related antibodies in renal tissues can directly trigger renal interstitial inflammatory responses, resulting in more severe interstitial edema and inflammatory cell infiltration, which causes inflammatory cells to leak through renal tubular epithelial gaps or damaged barriers, which requires further investigation. As is well known, hypocomplementemia is associated with cryoglobulinemia, and the decrease in complement observed in the SLE patients with cryoglobulinemia described in this article is also consistent with the results of two major previous studies [5,10]. Additionally, in our study, SLE patients mainly presented with mixed cryoglobulinemias, predominantly type III, which is consistent with previous research data on cryoglobulinemia in SLE [6,19,20]. In our cohort, patients with type II cryoglobulinemia demonstrated significantly higher serum creatinine and IgM levels compared to those with type III cryoglobulinemia. This discrepancy may be attributed to the fact that type II cryoglobulins are predominantly composed of monoclonal IgM or IgG. The monoclonal components in these cryoglobulins potentially exhibit enhanced binding affinity to autoantibodies, enabling the formation of stable immune complexes. These complexes subsequently deposit on glomerular capillary walls, triggering robust inflammatory cascades that exacerbate renal parenchymal damage and ultimately culminate in elevated serum creatinine levels ^[21]^. Additionally, the increased IgM levels could also be linked to clonal B-cell proliferation (including lymphoma-associated dysregulation). However, no cases of B-cell lymphoproliferative disorders were identified among the lupus nephritis patients complicated by type II cryoglobulinemia in this study. In patients with LN complicated by type II cryoglobulinemia, we also found that 2 patients were concurrently affected by Sjögren’s syndrome (2/9, 22%). Sjögren’s syndrome can also secondarily induce cryoglobulinemia through immune dysregulation. When LN is combined with Sjögren’s syndrome, excessive B-cell proliferation leads to the production of abnormal immunoglobulins (such as IgM with rheumatoid factor activity), which deposit in the kidneys and may exacerbate renal damage.

Histopathologically, 16.0% (13/81) of cryoglobulinemia patients exhibited glomerular electron-dense deposits with microtubular, fibrillary, fingerprint-like, and tubuloreticular ultrastructures under electron microscopy, predominantly localized in subepithelial and subendothelial regions. The remaining patients demonstrated comparable activity and chronicity indices by light microscopy. The finding might suggest that cryoglobulin could damage the kidney by activating the immune system. At the same time, some patients suffer double-shock from lupus and cryoglobulin independently. However, certain studies indicate that the presence of cryoglobulinemia does not exhibit a statistically significant correlation with renal involvement in SLE patients [5,10,21]. Since no associations were observed in our study, further research is needed to confirm its potential clinical significance. In our cohort, LN patients with cryoglobulinemia demonstrated no statistically significant differences in renal histopathological activity indices (both acute and chronic active lesions) compared to those without cryoglobulinemia. Clinically, aside from leukocyturia, no substantial discrepancies were observed in renal function parameters between the two groups. This finding suggests that the presence of cryoglobulinemia has little impact on the kidneys of SLE patients, with most being asymptomatic.

Given the relatively low incidence of SLE and the rarity of detected cryoglobulinemia cases, treatment protocols remain largely undefined. Current therapeutic approaches are tailored to the underlying etiology inducing cryoglobulinemia and the severity of clinical manifestations. In our cohort, patients with active SLE (SLEDAI-2K ≥ 6) universally received induction therapy with varying doses of glucocorticoids combined with immunosuppressants. No significant intergroup differences were observed in renal response rates or occurrence of composite endpoint events. Cox regression analysis indicated that cryoglobulinemia was not an independent risk factor for renal prognosis in patients with LN. However, due to insufficient sample size, further study needs to verify the findings. While therapeutic plasma exchange is theoretically capable of transiently removing circulating cryoglobulins and limiting their tissue deposition, the therapeutic benefits of this intervention in clinical practice have not been definitively established [22]. Despite the absence of randomized controlled trials (RCTs) evaluating PE for cryoglobulinemia, accumulating observational evidence from case series and uncontrolled studies supports its clinical utility in managing active moderate-to-severe cryoglobulinemia, particularly in critical scenarios involving renal compromise (including acute kidney injury), as an adjunctive intervention to mitigate irreversible renal damage [22–25]. However, in our matched cohort, cryoglobulinemia positive LN patients treated with PE combined with corticosteroids and immunosuppressive therapy showed no significant differences in renal remission or composite endpoint events compared to those who did not receive PE, suggesting that PE may not provide additional therapeutic benefits, but this cannot be ruled out as being due to limited sample size, and further studies are needed to verify this. These findings indicate that cryoglobulinemia does not exert a substantial impact on renal prognosis in LN. This is a retrospective study in which no quantitative cryoglobulin testing was performed. These are only our preliminary results, and further studies are needed to confirm. Therefore, clinical management of LN with concomitant cryoglobulinemia should prioritize optimizing conventional LN management strategies rather than targeting cryoglobulinemia specifically.

There are several limitations of our study. Firstly, this was a single-center retrospective study, and further studies with larger sample sizes and long-term follow-up could provide more definitive insights into this relationship. Secondly, the concentration of the cryoprecipitate was not detected for our laboratory techniques. Future studies will prioritize mechanistic investigations to elucidate the underlying pathophysiological mechanisms.

In conclusion, our study demonstrates that although cryoglobulinemia is relatively common in LN, the existence and types did not appear to significantly influence the renal outcomes. The treatment approach for LN patients with cryoglobulinemia should remain focused on managing underlying LN. In LN patients with cryoglobulinemia, no significant difference was observed in PE therapy, which still requires further research.

## Supplementary Material

SUPPLEMENTARY FILE.docx

## Data Availability

Clinical data involve patient privacy. The data used and analyzed in this study are available upon reasonable request from the corresponding author.
