# Supplementary material for: An observational study on lupus nephritis combined with cryoglobulinemia
Source: Ren Fail. 2026 Apr 6;48(1):2647568. doi: 10.1080/0886022X.2026.2647568 (PMC13059027; doi:10.1080/0886022X.2026.2647568)

**Supplementary table 1 Clinical features and prognosis of cryoglobulinemia with/without ultrastructural substructures**

|  | LN and cryoglobulinemia with **ultrastructural substructures**  (n=13) | LN and cryoglobulinemia without **ultrastructural substructures**  (n=68) | P value |
| --- | --- | --- | --- |
| Gender/Female, n (%) | 10 (76.9%) | 50 (73.5%) | 1.000 |
| Age at diagnosis of SLE, years | 26.0 (16.5, 29.5) | 30.0 (24.3, 45.5) | **0.024** |
| **Clinical manifestations** |  |  |  |
| Visual disturbance, n (%) | 0 | 3 (4.4%) | 1.000 |
| Arthritis, n (%) | 0 | 8 (11.8%) | 0.343 |
| Rush, n (%) | 3 (23.1%) | 20 (29.4%) | 0.749 |
| Alopecia, n (%) | 3 (23.1%) | 10 (14.7%) | 0.429 |
| Mucosal ulcers, n (%) | 0 | 5 (7.4%) | 0.587 |
| Fever, n (%) | 0 | 5 (7.4%) | 0.587 |
| Raynaud’s phenomenon, n (%) | 0 | 3 (4.4%) | 1.000 |
| NPSLE, n (%) | 0 | 0 |  |
| **Renal features** |  |  |  |
| Urinary casts, n (%) | 3 (23.1%) | 30 (44.1%) | 0.157 |
| Hematuria, n (%) | 12 (92.3%) | 51 (75.0%) | 0.278 |
| Proteinuria, n (%) | 13 (100%) | 63 (92.6%) | 0.587 |
| **Leukocyturia**, n (%) | 6 (46.2%) | 45 (66.2%) | 0.215 |
| AKD, n (%) | 1 (7.7%) | 24 (35.3%) | 0.055 |
| NS, n (%) | 9 (69.2%) | 37 (54.4%) | 0.323 |
| **Laboratory related tests** |  |  |  |
| WBC, 10^9^/L (3.5-9.5 10^9^/L) | 6.70 (4.48, 7.70) | 5.41 (3.80, 8.28) | 0.714 |
| Hb, g/L (115-150 g/L) | 114.8 ± 22.0 | 103.2 ± 21.3 | 0.077 |
| PLT, 10^9^/L (125-350 10^9^/L) | 183.0 (139.0, 269.0) | 190.5 (130.3, 265.5) | 0.954 |
| Alb, g/L (40-55 g/L) | 24.07 ± 6.86 | 27.77 ± 6.37 | 0.062 |
| Scr, μmol/L (44-133 μmol/L) | 65.3 (48.0, 116.3) | 96.1 (63.2, 170.3) | 0.060 |
| 24-hour urinary protein, g/day (0-0.15 g/24h) | 6.20 (1.82, 8.09) | 3.58 (1.87, 5.84) | 0.258 |
| C3, g/L (0.6-1.5 g/L) | 0.455 (0.311, 0.523) | 0.387 (0.303, 0.470) | 0.244 |
| C4, g/L (0.12-0.36 g/L) | 0.074 (0.048, 0.104) | 0.059 (0.035, 0.119) | 0.558 |
| IgG, g/L (7.23-16.85 g/L) | 11.10 (5.65, 15.65) | 12.10 (7.40, 17.28) | 0.666 |
| IgA, g/L (0.69-3.82 g/L) | 2.11 (1.51, 3,16) | 2.27 (1.79, 3.38) | 0.545 |
| IgM, g/L (0.63-2.77 g/L) | 1.00 (0.58, 1.30) | 0.91 (0.67, 1.42) | 0.949 |
| ANA | 13 | 68 | 1.000 |
| Anti ds-DNA antibody, n (%) | 7 (53.8%) | 51 (75.0%) | 0.177 |
| Anti-Sm antibody (+), n (%) | 3 (23.1%) | 15 (22.1%) | 1.000 |
| Anti-rRNP antibody(+), n (%) | 6 (46.2%) | 14 (20.6%) | 0.076 |
| SLEDAI-2K | 15.5 ± 3.3 | 16.9 ± 6.2 | 0.281 |
| **Renal pathology** |  |  |  |
| Class I, n (%) | 0 | 1 (1.5%) | 0.093 |
| Class II, n (%) | 1 (7.7%) | 2 (2.9%) |  |
| Class III (include III+V), n (%) | 0 | 13 (19.1%) |  |
| Class IV (include IV+V), n (%) | 8 (61.5%) | 45 (66.2%) |  |
| Pure Class V, n (%) | 4 (30.8%) | 7 (10.3%) |  |
| Activity indices score | 7.0 (1.0, 11.5) | 8.0 (5.0, 11.0) | 0.574 |
| Endocapillary hypercellularity | 3.0 (0, 3.0) | 3.0 (1.0, 3.0) | 0.840 |
| Glomerular leukocyte infiltration/Karyorrhexis | 1.0 (0.5 , 3.0) | 1.0 (1.0, 3.0) | 0.914 |
| Fibrinoid necrosis | 0 (0, 1.0) | 0 (0, 0) | 0.530 |
| Cellular/fibrocellular crescent | 0 (0, 2.0) | 2.0 (0, 2.0) | 0.350 |
| Platinum ear/microthrombosis | 0 (0, 3.0) | 1.0 (0, 3.0) | 0.245 |
| Interstitial inflammatory cell infiltration | 0 (0, 2.0) | 1.0 (0, 2.0) | 0.676 |
| Chronicity indices score | 2.0 (0, 2.5) | 1.0 (0, 2.0) | 0.795 |
| Glomerular sclerosis | 0 (0, 0.5) | 0 (0, 1.0) | 0.742 |
| Fibrous crescents | 0 (0, 0) | 0 (0, 0) | 0.167 |
| Tubular atrophy | 1.0 (0, 1.0) | 0 (0, 1.0) | 0.478 |
| Interstitial fibrosis | 1.0 (0, 1.0) | 0 (0, 1.0) | 0.890 |
| **Therapeutic options** |  |  |  |
| High-dose GCs, n (%) | 4 (30.8%) | 31 (45.6%) | 0.555 |
| CTX, n (%) | 7 (53.8%) | 39 (57.4%) | 0.948 |
| MMF, n (%) | 4 (30.8%) | 16 (23.5%) |  |
| CNIs, n (%) | 2 (15.4%) | 10 (14.7%) |  |
| Others (inclued LEF), n (%) | 0 | 3 (4.4%) |  |
| PE, n (%) | 0 | 7 (10.3%) | 0.591 |
| Rituximab, n (%) | 1(7.7%) | 1(1.5%) | 0.297 |
| Belimumab, n (%) | 2(15.4%) | 5(5.9%) | 0.245 |
| **Follow-up time, months** | 60.0 (49.5, 88.5) | 61.5 (42.0, 88.0) | 0.743 |
| **Renal remission status** |  |  |  |
| CRR, n (%) | 8 (61.5%) | 39 (57.4%) | 1.000 |
| PRR, n (%) | 3 (23.1%) | 18 (26.5%) |  |
| NR, n (%) | 2 (15.4%) | 11 (16.2%) |  |
| **Long-term survival** |  |  |  |
| Died, n (%) | 2 (15.4%) | 11 (16.2%) | 1.000 |
| ESKD, n(%) | 0 | 4 (5.9%) | 0.591 |

Abbreviations: LN, lupus nephritis; SLE, systemic lupus erythematosus; NPSLE, neuropsychiatric systemic lupus erythematosus; AKD, acute kidney disease; NS, nephrotic syndrome; WBC, white blood cell; Hb, Hemoglobin; PLT, Platelet; Alb, albumin; Scr, serum creatinine; C3, Complement 3; C4, Complement 4; IgG, **Immunoglobulin G;** IgA, **Immunoglobulin A;** IgM, **Immunoglobulin M; ANA, antinuclear antibody;** Anti ds-DNA, a**nti-double-stranded DNA antibody;** Anti Sm, anti-smith antibody; anti rRNP, anti-ribonucleoprotein antibody; SLEDAI-2K, systemic lupus erythematosus disease activity index 2000; APS, antiphospholipid syndrome; GCs, glucocorticoids; CTX, cyclophosphamide; MMF, mycophenolate mofetil; CNIs, calcineurin inhibitors; LEF, leflunomide; PE, plasma exchange; CRR, complete renal response; PRR, partial renal response; NR, no response; ESKD, end stage kidney disease.

**Supplementary table 2-1 Univariate and Multivariate Cox regression analysis of risk factors for renal prognosis in LN patients**

| Variables | Univariate analysis | | Multivariate analysis | |
| --- | --- | --- | --- | --- |
|  | HR (95%CI) | P value | HR (95%CI) | P value |
| Female | 1.654 (0.854-3.202) | 0.136 |  |  |
| Age | 1.011 (0.986-1.037) | 0.406 |  |  |
| With cryoglobulinemia | 1.004 (0.498-2.021) | 0.992 |  |  |
| Proteinuria | 2.919 (1.016-8.389) | **0.047** | 10.815 (2.793-41.875) | **<0.001** |
| **Leukocyturia** | 2.336 (1.206-4.526) | **0.012** |  |  |
| IgA, g/L | 0.643 (0.468-0.884) | **0.007** |  |  |
| Anti ds-DNA (+) | 4.029 (2.038-7.967) | **<0.001** | 4.700 (1.918-11.515) | **<0.001** |
| Chronicity indices score (CI) | 1.475 (1.229-1.770) | **<0.001** |  |  |
| Glomerular sclerosis | 3.155 (1.929-5.518) | **<0.001** |  |  |
| Tubular atrophy | 1.837 (1.167-2.894) | **0.009** |  |  |
| Interstitial fibrosis | 2.113 (1.230-3.630) | **0.007** |  |  |
| SLEDAI-2K | 0.947 (0.897-0.999) | **0.046** |  |  |
| With PE | 0.248 (0.101-0.607) | **0.002** |  |  |

Abbreviations: HR: hazard ratio; CI: confidence interval; IgA, **Immunoglobulin A;** Anti ds-DNA, a**nti-double-stranded DNA antibody; SLEDAI-2K, systemic lupus erythematosus disease activity index 2000;** PE, plasma exchange.

**Supplementary table 2-2 Refitted Cox Regression Results After LASSO-Cox Variable Selection (λ_min)**

| Variables | β | HR | 95% CI | P |
| --- | --- | --- | --- | --- |
| Proteinuria | -1.941 | 0.144 | 0.045–0.462 | 0.001 |
| **Leukocyturia** | -0.536 | 0.585 | 0.289–1.186 | 0.137 |
| IgA | -0.251 | 0.778 | 0.579–1.044 | 0.094 |
| Anti ds-DNA (+) | -1.303 | 0.272 | 0.121–0.609 | 0.002 |
| Chronicity indices score (CI) | 0.230 | 1.258 | 0.961–1.647 | 0.095 |
| Glomerular sclerosis | 0.603 | 1.828 | 0.854–3.911 | 0.120 |
| With PE | 0.465 | 1.593 | 0.586–4.326 | 0.361 |

Note: n=127, events=38; λ_min=0.074425. First, variables were selected using LASSO-Cox (10-fold cross-validation) at λ_min. Then, the conventional Cox proportional hazards model was re-fitted with the selected variables, and the hazard ratios (HRs) and 95% confidence intervals (95% CIs) were reported.

**Supplementary figure 1. The enrolment of lupus nephritis patients**


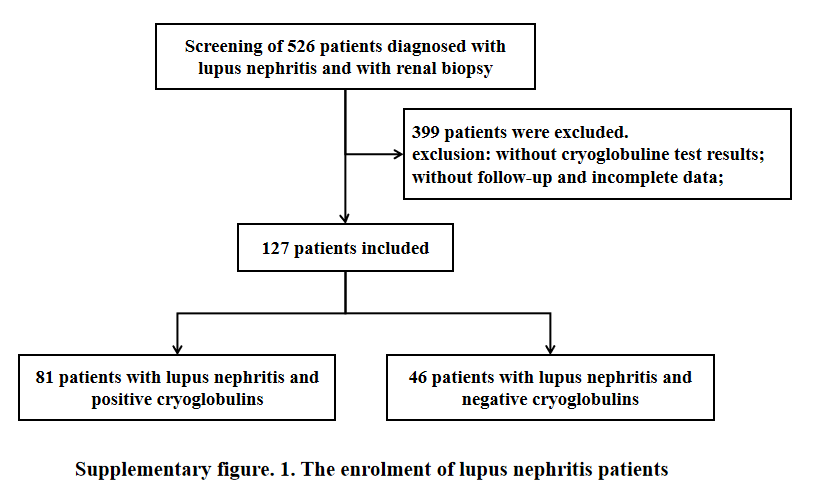

Supplement: SUPPLEMENTARY FILE.docx [file IRNF_A_2647568_SM2155.docx]
